# Telemedicine Use Among Caregivers of Cancer Patients: Systematic Review

**DOI:** 10.2196/jmir.9812

**Published:** 2018-06-18

**Authors:** Chiara Marzorati, Chiara Renzi, Samuel William Russell-Edu, Gabriella Pravettoni

**Affiliations:** ^1^ Applied Research Division for Cognitive and Psychological Science European Institute of Oncology Milan Italy; ^2^ Department of Oncology and Hemato-Oncology University of Milan Milan Italy; ^3^ Library European Institute of Oncology Milan Italy

**Keywords:** telemedicine, family, caregivers, neoplasms, systematic review

## Abstract

**Background:**

The number of published studies and systematic reviews examining different telehealth interventions targeting patients and their effects on patients’ well-being and quality of life have grown in recent decades. However, the use of telemedicine tools aimed at the family members and caregivers of adult cancer patients is less defined.

**Objective:**

We aimed to conduct a systematic review to provide a more complete picture regarding telemedicine tools for informal caregivers (usually family members or close friends) implemented in all phases of cancer care. More specifically, the review aimed to better describe the study samples’ characteristics, to analyze measured outcomes and the specific questionnaires used to assess them, and to describe in depth the implemented interventions and their formats. Finally, we examined the role of telehealth, and usability and feasibility trends in supporting patients’ caregivers.

**Methods:**

We systematically searched the literature in the following databases: Web of Science, Cochrane Library, PubMed, Scopus, CINAHL, MEDLINE, EMBASE, Google Scholar, and PsycINFO. Inclusion criteria were being written in English, published in peer-reviewed journals, describing a telehealth-implemented intervention, and focusing on caregivers of adult cancer patients at any stage of the disease. We selected studies published up to November 2017. We critically appraised included articles using the Preferred Reporting Items for Systematic Reviews and Meta-Analyses and graded the quality of evidence by outcome using the Centre for Evidence-Based Medicine framework.

**Results:**

We included 24 studies in the final selection. In 21 of the 24 studies, the patient-caregiver dyad was analyzed, and the study population dealt with different types of cancer at different stages. Included studies considered the caregiver’s condition from both an individual and a relational point of view. Along with psychosocial variables, some studies monitored engagement and user satisfaction regarding Web-based platforms or telehealth interventions. All studies reported significant improvements in some of the investigated areas, but they often showed small effect sizes. Two types of telehealth intervention formats were used: Web-based platforms and telephone calls. Some of the included studies referred to the same project, but on study samples with different cancer diagnoses or with new versions of previously developed interventions.

**Conclusions:**

Reported outcomes seem to suggest that we are in an exploratory phase. More detailed and targeted research hypotheses are still needed. Clarifying caregivers’ needs related to telehealth tools and better defining outcome measures may yield more significant results.

## Introduction

### Telemedicine Overview

Information and communication technology (ICT) has in recent decades become essential in supporting information provision, sharing data, overcoming face-to-face boundaries, and meeting people’s needs [1]. In the medical field, transferring information through telecommunication networks easily provides an opportunity for innovation, and helps in managing resources and increasing health care quality.

The use of medical information exchanged from one site to another via electronic communications to improve a patient’s clinical health status is defined as telemedicine [2]. It includes a variety of applications that allow the transfer of eHealth data. eHealth systems allow many different hospital facilities to cooperate to improve health care services, patient engagement, monitoring, and management, and to provide prompt access to expert advice and patient information, regardless of where patients are or where information is collected. From this perspective, ICT may support the global drive to achieve consistent, integrated, sustainable, high-quality, and cost-efficient health care [3,4]. Web-based interventions present innovative methods for using and improving public health services with easily accessible, up-to-date, and tailored information, education programs, self-management training and monitoring, and family-physician communication [5-11].

To date, eHealth interventions have mostly been implemented to support the self-management of “the big five” diseases identified by the World Health Organization, namely diabetes mellitus, cardiovascular and chronic respiratory diseases, cancer, and stroke [12]. A scoping review showed how people affected by chronic conditions used ICT especially for self-management, thus enhancing patient engagement. The broadest category where ICT interventions were implemented was cancer care, with specific focus on shared management activities among patients and their providers [13].

### Telemedicine for Caregivers of Cancer Patients

Alongside the development of telehealth interventions aimed at cancer patients, attention is increasingly being directed toward telemedicine tools aimed at satisfying the needs of caregivers.

Caregivers are usually family members or close friends whose efforts to care for their loved ones have a considerable physical and psychological impact on them. Family members are often considered fundamental in the process of care, especially for those diseases that require continuous or extended treatments. Demographic and health trends among the European population are increasing the need for reorganizing and delivering better and more cost-effective health services [14], not only for patients but also for caregivers. Caregiver care has thus become a core topic of contemporary scientific research because it can be related to prevention: if more attention and assistance is given to caregivers, they will experience fewer physical and psychological impairments, thereby having less of an impact on the health care system from an economic or a social perspective [15,16]. Literature reviews and meta-analyses confirm the association between greater mental burden and poorer physical and mental well-being: responsibilities and stressful experiences related to the caregiving role can lead to depression, anxiety, worry, and loneliness [17-22]. Similarly, the greater mental burden and emotional distress caregivers experience can result in fatigue, sleep impairment, and unhealthy behaviors [23-26]. Several studies have demonstrated highly distressing conditions among caregivers, affecting them not only psychologically but also physiologically. Depression, anxiety, or poor sleep quality can cause a decline in immunocompetence and can be associated with the onset of cardiovascular disease or earlier death [27-29]. The physical and psychological impairments of caregivers are well documented in the field of oncology; poorer physical health of cancer patients is significantly associated with a deterioration of physical health among family members [30], as well as with symptoms of depression or anxiety [31,32]. Providing cancer care for years or resuming care before the patient’s death can also be related to the emergence of arthritis, heart diseases, and chronic back pain in the caregiver [33]. These studies have shown how cancer caregiving is highly demanding and emotionally burdensome, leading to the need for information to manage patients’ symptoms or improve knowledge in medical procedures. Longacre [34] classified caregivers’ information needs into personal psychosocial care, the provision of direct care, and care management. She pointed out that meeting those needs positively interacts with caregivers’ perception of managing emotional and physical stress. Another systematic review [35] showed how needs were unmet mostly in terms of diagnosis- and prognosis-related information, information about the impact on the family or partner, information on practical issues, coping information, and medical information. Caregivers also asked for support for their psychological condition and their fears concerning the patient’s disease progression or recurrence [36].

The possibility of creating new direct and interactive interventions—directed not only at patients, but also at caregivers—places greater attention on eHealth tools in the context of long-term diseases.

Caregivers, as well as patients, are increasingly using apps and Web-based interventions to cope with their uncertainty and need for information. Caregivers need to be informed about and prepared for patient symptoms or side effects, and they want better knowledge to counter their fears of inadequacy, for example [37,38].

Even though much has been done concerning patient empowerment, more attention needs to be paid to the effects and support of telemedicine on family caregivers and on how promising eHealth programs are in responding to their needs [39,40]. Despite caregivers’ requests for provision of support and information competence, a recent meta-review on the effects of eHealth for cancer patients and caregivers concluded that there is indeed a paucity of systematic reviews on this topic and that Web-based interventions focused on family members are still an unexplored area [41].

The number of published studies and systematic reviews examining many types and effects of Web-based interventions targeting patients have increased in recent decades [42-44]. However, less is known about telehealth interventions aimed at the cancer patient’s family members. Scoping reviews have been conducted only on Web-based interventions or on the effects of eHealth tools for cancer patients and their informal caregivers [41,45,46], while others had a broader focus on all implemented telehealth tools for family caregivers, but not specifically involved in cancer care [39]. Therefore, the need for obtaining a more complete picture of implemented telemedicine tools for caregivers in all phases of cancer care is emerging.

### Objectives

This systematic review aimed to describe the main characteristics of previously developed telehealth tools for family members of cancer patients. More specifically, the objectives of the study were to better describe the samples’ characteristics, to specify the measured outcomes and the specific questionnaires used to assess them, and to describe in depth the implemented interventions and their formats. Alongside the implementation of telemedicine systems for caregivers, we hoped to identify the main considered outcomes, to analyze the role of eHealth technology, and to discuss the usability and feasibility trends in supporting patient caregivers.

## Methods

We conducted a systematic review of studies on telehealth-based intervention for caregivers of cancer patients at any stage of the disease.

### Search Strategy

We systematically searched the following databases: Web of Science, Cochrane Library, PubMed, Scopus, CINAHL, MEDLINE, EMBASE, Google Scholar, and PsycINFO.

We used various combinations of database-specific controlled vocabularies (subject headings), supplemented by keywords, and title and abstract terms for the concepts and synonyms relating to telemedicine, telehealth, Web-based intervention, eHealth, mHealth, carers, caregivers, family, and cancer. We examined bibliographies and reference lists of relevant articles and identified citing articles using Web of Science. No time restrictions were applied. English language restriction was applied. Multimedia Appendix 1 reports the full search strategies we used.

### Selection Strategy

One of the authors (SWRE), a qualified medical librarian, conducted the systematic literature search. Two other authors (CR and CM) selected articles for full review based on the inclusion and exclusion criteria and assessed their eligibility. Agreement was reached on the final selection of included studies.

For study inclusion in this systematic review, we applied the following selection criteria: (1) written in English, (2) published in peer-reviewed journals, (3) including a telehealth-implemented intervention (4) involving human participants, and (5) focusing on caregivers of adult patients at any cancer stage. We excluded studies that did not involve human participants or did not have an experimental study design (eg, commentary, review, or expert opinions). We selected studies published up to November 2017.

### Review Strategy and Data Extraction

The initial search resulted in 655 articles. We also searched the reference lists of relevant articles to identify other articles. We excluded 413 articles based on a review of titles and keywords. Subsequently, we excluded 170 articles based on their abstracts because they did not meet the inclusion criteria. After eliminating 48 duplicates, we included 24 studies in the final selection.

**Figure 1:**
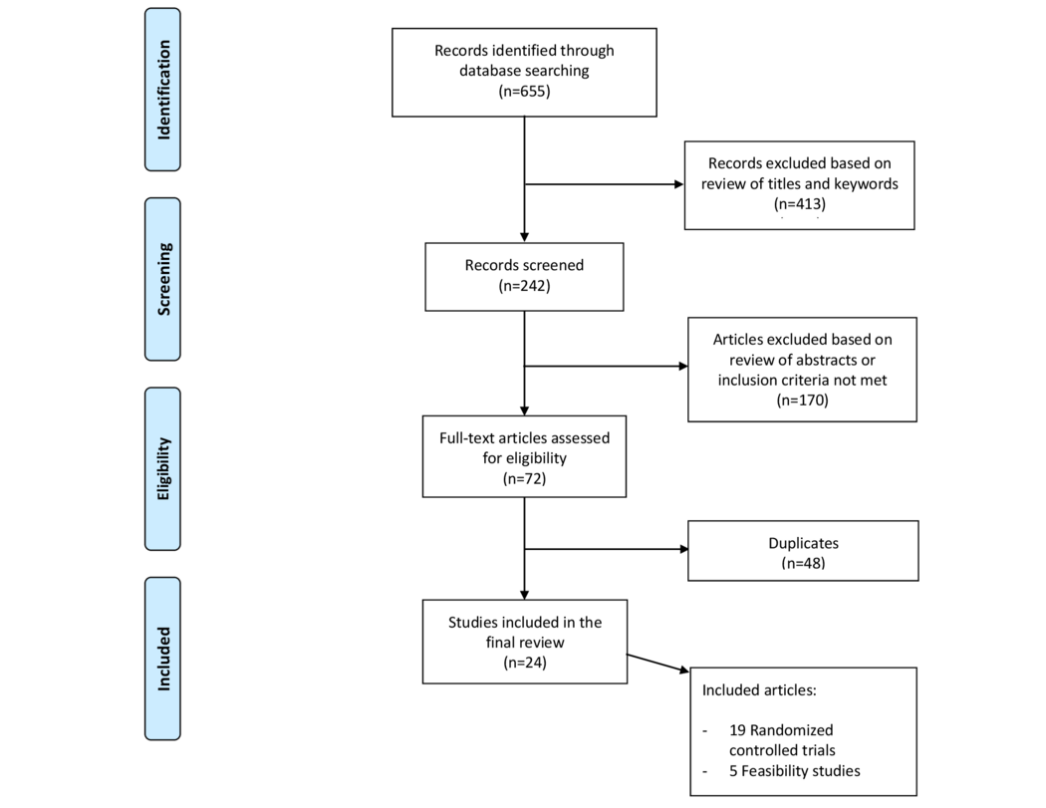
Preferred Reporting Items for Systematic Reviews and Meta-Analyses (PRISMA) flowchart.

We applied the Preferred Reporting Items for Systematic Reviews and Meta-Analyses (PRISMA) guidelines. Figure 1 shows the PRISMA flowchart.

To evaluate the strength of the studies’ findings, we also scored each article for the level of evidence according to the Centre for Evidence-Based Medicine framework: 1a: meta-analyses; 1b: individual randomized controlled trials (RCTs); 1c: non-RCTs; 2a: systematic reviews of cohort studies; 2b: individual cohort studies; 2c: outcomes research; 3a: systematic reviews of case-control studies; 3b: individual case-control studies; 4: case series; and 5: expert opinions without explicit critical appraisal [47].

We used a standardized form for data extracted from the included articles, outlining the year of publication, authors, study country, aim, sample characteristics, study design, type of intervention, measured outcomes, assessment, and principal results.

We grouped the included studies into 3 subcategories according to which kind of intervention was implemented: eHealth intervention, telephone sessions, or both. We divided the measured outcomes into clinical and usability subgroups, then split clinical outcomes into psychosocial (in turn divided into the caregiver’s individual and dyadic dimensions) and behavioral factors.

**Figure 2:**
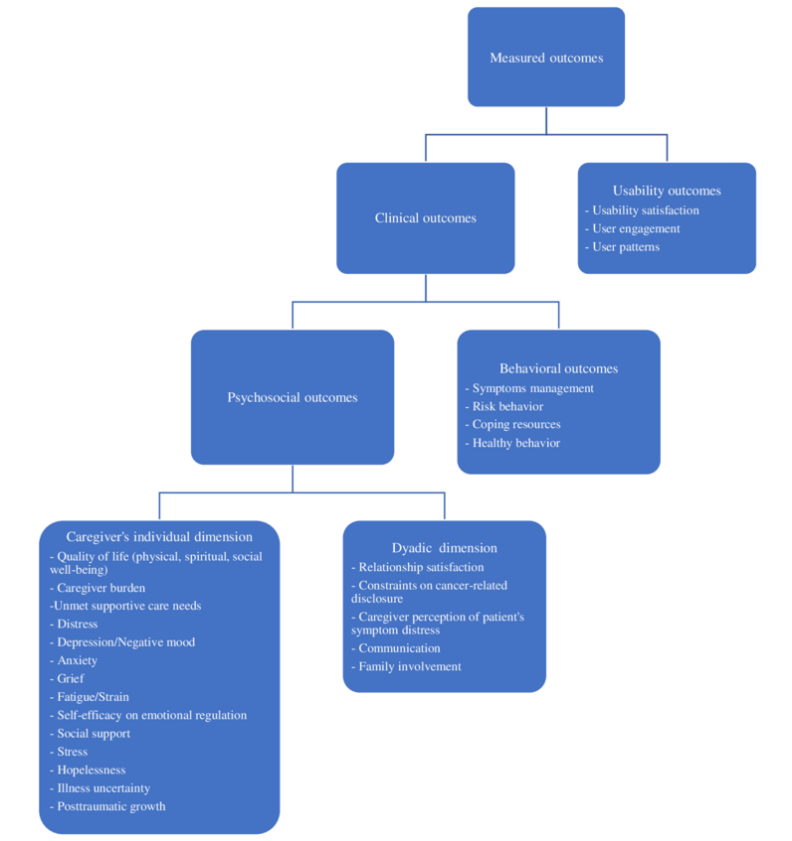
Categorization of measured outcomes in the 24 reviewed articles.

Figure 2 shows the categorization of the measured outcomes.

## Results

### Study Designs

Study designs comprised 19 RCTs (1b) and 5 feasibility studies (1c). Of the RCTs, 16 had 2 arms, whereas the remaining 3 had 3 arms; 7 did not include a no-treatment control group. All RCTs provided follow-up assessment at 3 and 6 months at least.

### Caregiver Characteristics

**Figure 3:**
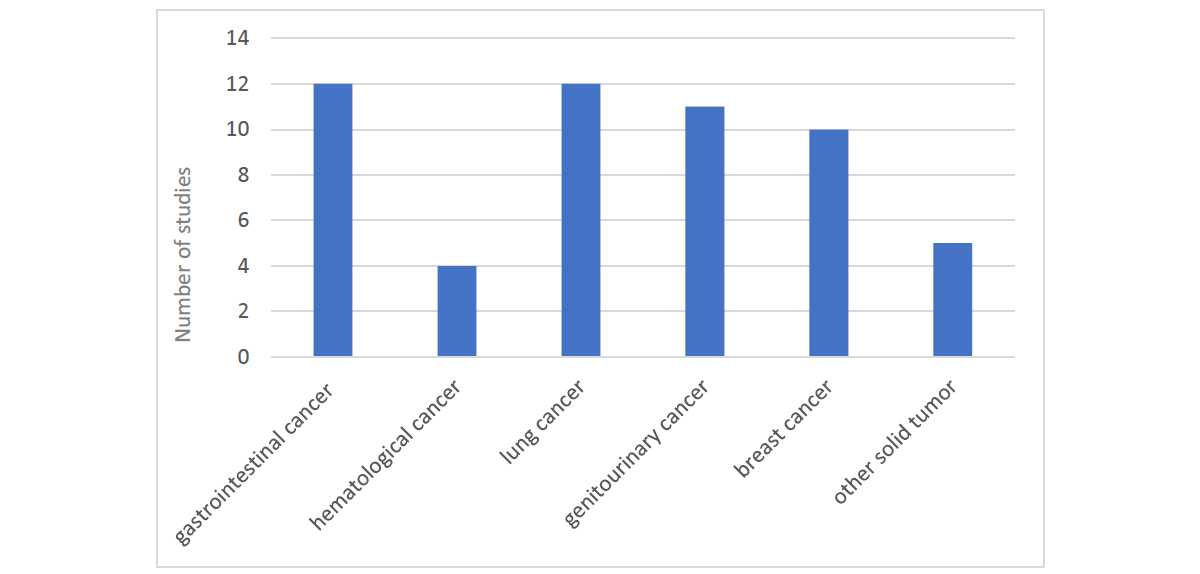
Cancer diagnoses considered in the 24 reviewed articles.

A total of 3301 caregivers of cancer patients were enrolled, with the number of family caregivers in each study ranging from 6 to 481 (see Multimedia Appendix 2 [48-71]). Of the 24 studies, only 1 did not provide sufficient data on their sample’s sex and age statistics (N=12) [48]. Of the 3289 remaining family members, 70.75% (2327/3289) were female and 56 years old on average. In 21 of the 24 studies, the patient-caregiver dyad was analyzed; interventions were delivered to both patients and caregivers, and outcomes were measured in both populations. In 3 studies, caregivers were the only target sample and patients variables were not considered [49-51]. A total of 3 studies focused the intervention only on women, 2 studies examined partners [52,53], and 1 included caregivers with different relationships to patients [54]. In many studies, patients identified not only their partners as their main caregivers (2590/3301, 78.46%), but also adult children, siblings, parents, or friends. Dyads dealt with different types of cancer at different stages; among the included studies, lung and gastrointestinal cancers were the most considered, followed by genitourinary and breast tumors, and hematological neoplasms (see Figure 3). Of the 24 studies, 8 focused on advanced cancers [48,51,54-59] and 2 on early-stage tumors [60,61]; the other trials included patients with all stages of the disease.

Study attrition rate varied from 3% to 64%. The main reasons for withdrawal were patient death, lack of interest, and medical condition (eg, progression of the disease).

Most of the studies were conducted in the United States (see Multimedia Appendix 2), except for 3 studies carried out in Australia [50,62], 1 in Sweden [63], and 1 in Canada [48].

### Measured Outcomes

Clinical and usability outcomes were measured in 7 studies [48,50,58,63-66]; in the remaining studies, only the first cluster was included. The caregiver’s well-being was assessed considering various psychosocial and behavioral variables, except for 2 studies that used a single outcome measure. Kinney et al [49] studied only change in colonoscopy prevention behavior, and Clark et al [67] assessed quality of life among caregivers of patients with advanced cancer undergoing radiotherapy treatment (see Multimedia Appendix 2). Other studies evaluated several outcomes, ranging from 2 [55,57] to 12 [59].

The 2 most-assessed behavioral outcomes were coping resources and symptom management. Coping resources were assessed either by the Brief COPE questionnaire [54,58] or by combining multiple questionnaires: the Lewis Mutuality and Interpersonal Sensitivity Scale, the Brief version of the Social Support Scale, and the Lewis Cancer Self-efficacy Scale [52,59,68]. Symptom management, on the other hand, was assessed only by qualitative analysis on audio-recorded interviews [65,69].

We created 2 categories of psychosocial outcome measures: one cluster included the caregiver’s individual dimensions, and the other cluster was related more to dyadic interactions (eg, relationship with partner, perception of patient’s health condition). In the first category, the most examined constructs were quality of life [50,52,54,56,59,62,66-68] and distress [50,52,53,59,61,62,64,68,70,71]. Quality of life was assessed with the Functional Assessment of Cancer Therapy-General (FACT-G; n=4), the Short Form Survey 36-item and 12-item versions (n=1), the Caregiver Quality of Life Scale-Cancer (n=2), the European Organisation for Research and Treatment of Cancer Quality of Life Core Questionnaire (EORTC QLQ-C30; n=1), and the Medical Outcomes Study 12-item Short Form Survey (n=1). Questionnaires used to measure the distress or stress condition were the Profile of Mood States (n=2), the General Symptom Distress Scale (n=2), the 77-item Omega Screening Questionnaire (n=2), the Distress Thermometer (n=1), the Brief Symptom Inventory 18 (n=1), the Perceived Stress Scale (PSS; n=1), and the Posttraumatic Stress Disorder Symptom Scale to measure cancer-specific distress (n=1). In addition, the caregiver’s depression [50,56,57,60,64,70], social support [58,59,64,66,68,70], and self-efficacy [52,59,61,68,69] were taken into account in almost one-third of the trials. Anxiety [69,70], hopelessness [52,54,59], fatigue [61,64,70], cancer knowledge [52,54,59,68], spiritual well-being [60,64,70], and uncertainty [52,54,59,65]were also measured in several studies (see Multimedia Appendix 2).

The second cluster, concerning the caregiver’s relationship with the patient, evaluated perceived support such as difficulties encountered in communicating with the patient about the disease and the caregiver’s perception of the patient’s symptom management [51,55,69]. The Edmonton Symptom Assessment Scale was the main tool used to assess these variables.

Along with clinical variables, some studies monitored engagement and user satisfaction regarding the Web-based platforms or telehealth interventions. User satisfaction and device usability were explored through open-ended questions [60,65,66], single-item questions such as “How comfortable are you using the internet?” [58], or semistructured interviews [48,50,63] that were audio recorded, transcribed verbatim, and coded with latent content analysis.

**Figure 4:**
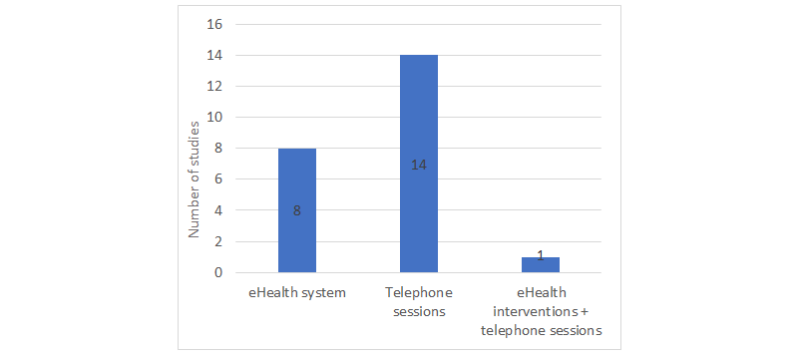
Intervention formats.

All studies reported significant improvements in some of the investigated areas, but they often showed small effect sizes. Even though statistically significant outcomes differed among the included studies, some of them were significant in more than 1 study measuring that specific outcome. These improved outcomes were caregiver self-efficacy, quality of life, distress, depression, appraisal of caregiving, and perceived social support. Caregiver self-efficacy, both in managing one’s own emotions and in helping patients to control symptoms, was measured in 5 studies [52,59,61,68,69] and was always statistically significant. A total of 5 studies reported significant differences in quality of life: 3 showed significant effects in all dimensions of quality of life [52,64,68]; 2 studies showed significant effects in spiritual and social well-being [59,64]; and 1 study [60] showed significant outcomes in social well-being. Emotional distress or stress symptoms were significantly different between pre- and posttreatment assessments in 5 of the considered studies [50,52,64,68,71], while both depressive symptoms [55,57,64] and perceived social support [62,64,66] improved in 3 trials. Furthermore, the included articles reported significant, though smaller, effects in other measured outcomes: after completing the interventions, family members also experienced less anxiety, less sense of disruptiveness, hopelessness, and uncertainty, less burden, and negative appraisal of caregiving.

### Technology Use and Intervention Format

Different telehealth interventions were conducted in the analyzed studies. Multimedia Appendix 3 provides a full description of the interventions delivered in each study.

In 12 studies, an intervention was developed for a sole cancer type [49,51-54,58,60,61,64,66,69,70]; the other studies implemented telemedicine tools for a multifaceted sample including patients with different cancer diagnoses [48,50,55-57,59,62,63,65,67,68].

A total of 2 studies, 2 conducted with a qualitative methodology [65] and 2 with a mixed-methods methodology [48], were scheduled for a one-time-only data collection, unlike all other trials, in which at least two follow-ups were planned after study completion.

The included studies used various telehealth intervention formats: some studies involved the development of a Web-based platform, while others used scheduled telephone calls to improve the dyads’ psychosocial condition. More precisely, 8 studies [50,51,55,58,63,65,66,68] implemented eHealth interventions aimed at exploring caregiver coping strategies, emotional well-being, and patient symptom management. Schover et al [53] combined the eHealth system with telephone sessions. All other studies used only telephone calls with supplemented written material to support patients and caregivers in their process of care (see Figure 4).

Of the 20 studies that provided for the presence of a practitioner to conduct part of the described interventions, 8 were conducted by trained nurses [48,52,54,56,59,61,62,65], 4 were carried out by social workers [60,64,69,70], and 3 were conducted at a distance by caregivers using Web-based platforms [51,55,58]. In 3 RCTs the intervention was delivered by clinical psychologists [53,62,71], and in another trial a genetic counsellor conducted the telehealth risk communication to promote colonoscopy screening [49]. Another 2 studies provided psychosocial support via a multidisciplinary team including a chaplain, counsellor, dietician, physiotherapist, and physician [63], or a clinical psychologist, psychiatrist, advanced practice nurse, hospital chaplain, clinical social worker, physiatrist, and physical therapist [67]. Master’s-level nurses, clinical social workers, and psychologists were trained, and sometimes allocated to multidisciplinary teams, to help caregivers in their process of patient care. All participants in both the experimental and the control groups received the telehealth intervention, except in 7 studies, where the control group received only usual care [49,52,54,59,62,66,67].

Some of the included studies referred to the same project but referred to population samples with a different cancer diagnosis or with new versions of previously developed interventions. Dionne-Odom et al [56,57] published 2 articles on patients with advanced cancer and their caregivers under the third Educate, Nurture, Advise Before Life Ends project; and 3 studies [51,55,58] were conducted within the Comprehensive Health Enhancement Support System program. The family involvement, optimistic attitude, coping effectiveness, uncertainty reduction, and symptom management (FOCUS) Program comprised 5 studies [52,54,59,65,68], and Badger et al conducted 3 RCT studies on breast [60,70] and prostate [64] cancer patients and their partners to examine the effectiveness of 2 different telephone interventions.

## Discussion

### Principal Findings

We systematically reviewed 24 studies implementing telehealth tools for caregivers of cancer patients. Interventions used telephone calls or eHealth systems aimed at improving the physical and mental well-being of the study populations to satisfy different user needs.

In this systematic review, only one-third of the studies used eHealth systems to investigate psychosocial outcomes. All the studies using Web platforms were published after 2011, except for 1 article published in 2002 [66], reflecting the rapid development of eHealth technology over the last few years. Most of the included studies instead implemented a supportive or educational intervention based on scheduled telephone calls and the distribution of written material. Badger et al [60] investigated users’ preferences for telephone, videophone, or face-to-face methods: dyads agreed that the telephone intervention (69% of patients and 73% of caregivers) was the most reliable and easy-to-use system, compared with a videophone intervention, which in turn was preferred to face-to-face interaction. Only 1 study [53] used both an eHealth system and telephone sessions, even though they were not compared, but formed part of the same intervention. The proportion of participants favoring telephone-delivered interventions over eHealth interventions suggests that the implementation of Web-based platforms in health care systems is still in development and that further research is needed.

Web-based interventions facilitate participant enrollment and data collection from patients, reduce the risk of missing items, overcome geographic and mobility problems, and are more cost effective, but researchers have less control over participants [72-75].

Telephone-based interventions, on the other hand, include personalized therapist guidance. This can positively influence patient outcomes and proactively support potential crises by virtue of a more individualized and tailored intervention than that delivered by telehealth programs [76]. These telephone sessions may also contribute to enhancing the sense of independence and autonomy for patients and caregivers [77]. However, published studies directly comparing internet-based versus telephone intervention are lacking. The use of Web-based platforms may thus reflect the shift in social and cultural trends related to the use of ICT rather than an actual evidence-based advantage of Web-based over telephone interventions [78].

In addition to the variability in telehealth interventions, there was also a consistent variability in the considered outcome measures used in the studies. In 24 articles, 30 different caregiver outcomes were measured to evaluate the interventions, with most being psychosocial variables. These psychosocial constructs were measured using a variety of questionnaires. For instance, studies considering quality of life as an outcome used either the 12-item Short Form Survey, the FACT-G, or the EORTC QLQ-C30 as a measurement tool. This finding is consistent with other studies assessing the effects of supportive telehealth interventions on psychosocial variables: Agboola et al [79] found a nonuniformity in measured outcomes and questionnaires assessing quality of life, depression, and pain management in patients with cancer.

Considering this, it could be interesting to identify the main tools and variables on which to focus. This would enable us to better define the theoretical framework within which the study programs are developed and to analyze results, and thus to disentangle explanatory relations between different variables. According to a literature review on telehealth interventions [80], a precise structure linking all aspects of the intervention or of the outcomes is rarely used, and most of the projects lack a theoretical framework. Nevertheless, even in the general literature (beyond that relating to telehealth interventions), there are unfortunately only a few studies that assessed correlations or mediated effects between different constructs (eg, quality of life, depression, and self-efficacy) that are related to caregivers’ needs (eg, information provision, social support, and self-management education) [81,82].

In most of the projects, telehealth tools were considered as a given: most of the studies focused on the efficacy of the tool to promote caregivers’ well-being, overshadowing the usability and feasibility of the eHealth programs. The accessibility and usability of the technology have not often been assessed, and only 6 of the 24 studies assessed user satisfaction or Web-based program usage patterns [48,50,58,60,63,65,66]. The lack of investigation in this area may prevent a correct evaluation of and improvement in the effectiveness of the implemented telehealth tools. In fact, without assessing all the included aspects of the effectiveness concept (user satisfaction, usefulness, interaction quality, and ease of use), it would be more difficult to understand whether it is the specific tool that does not function or whether it could even be telehealth interventions in general [83,84].

Different aspects of each intervention may benefit to a greater or lesser extent from using an eHealth delivery. For instance, using eHealth may enable screening for aspects of caregivers’ well-being, which may otherwise remain unconsidered due to lack of resources or due to inefficiency in standard care flows.

In accordance with other literature reviews and meta-analyses [41,85], our review found that family members who used telehealth tools reported a perception of increased social support [58,60,64,68] and a less negative appraisal of illness and caregiving [52,54,68], even though the overall effect sizes were small. These findings meet Kent and colleagues’ research recommendations to improve the assessment of the prevalence and burden of informal cancer caregiving [86], emphasizing the need to direct attention toward the most vulnerable caregivers of cancer patients, such as those socially isolated, living in rural areas, or with low socioeconomic status. Social isolation and low appraisal of caregiving, along with depression, financial stress, and lack of choice in being a caregiver, are important risk factors of caregiver strain, affecting their perception of burden [87,88]. Therefore, understanding the impact of caregiving and developing tailored interventions to provide assistance to caregivers can satisfy important unmet needs and reduce caregivers’ psychological and emotional burden [89].

In some cases, different studies of the same project reported conflicting results on psychological outcomes. For instance, Badger et al [60] found significant improvements in perceived social well-being in the telephone and videophone interpersonal counselling group, but not in the health education group. This contrasts with 2 years previously, when they reported greater improvements in the health education group than in the videophone counselling group [64] in the same variable. This variability may be related to the enrollment of individuals in different disease stages, undergoing different treatments, or having different psychological and social characteristics, with these differences applying to both the patient and the caregiver. It is known that different aspects of the disease or of the treatment may imply different caregiving burdens [41], as well as different psychological or relational issues [90-94]. Individuals may be more or less able to manage the demands related to caregiving, depending on, for example, their socioeconomic status, literacy level, personality traits, resilience, and contingent factors [95,96]. It follows that it may be necessary to compare the same intervention across different caregiver populations and, further, to personalize the intervention depending on aspects that turn out to be significant in determining the outcome(s).

While usability testing and psychological variables have been sufficiently, though not equally, considered, studies are lacking that assess the specific dimension related to changes in the caregivers’ perception of their role. More precisely, studies did not include specific measures to detect differences in the caregivers’ appraisal of patient management after using telehealth interventions. For example, it is difficult to disentangle whether and to what extent changes in caregivers’ self-efficacy were directly related to use of the tool, since most of the studies had no control group. So far, the design of the studies has not allowed for evaluation of whether the use of the tools (dose, frequency, or satisfaction) was a mediator of the outcome (eg, self-efficacy).

It is clear that telehealth implies not only the mere use of electronic services to store medical data, but also a more complex framework. This includes practitioners’ education, patients’ and caregivers’ empowerment, efficiency, equity, quality of service provision, and promotion of shared decision processes at local, national, and global health care levels [97].

It would be interesting to reconsider future directions: reported outcomes seem to suggest that we are in an exploratory phase. There is still a need to construct more detailed and targeted research hypotheses. The lack of theoretical frameworks leads to the implementation of broad yet possibly weak interventions, targeting many different constructs or aspects, and thus may lead to nonsignificant results or to small effect sizes. Clarifying caregivers’ needs related to telehealth tools and better defining outcome measures may allow us to obtain more significant results.

### Conclusion

It is crucial to identify unmet family needs or priority clusters and to take into account the relation to cost-effectiveness trends. There is a paucity of studies assessing the economic value of psychosocial interventions with standardized methods [98,99]. Future studies can provide further cost-related information to support decision-making processes and the planning of new large-scale care services. To achieve value-based health care, it is important to devise cost-effective study designs and to implement the most appropriate data collection methods and procedures in order for the results to be generalizable across different populations and contexts [100].

## Appendix

Multimedia Appendix 1 Search strategy.

Multimedia Appendix 2 Details of the included studies.

Multimedia Appendix 3 Formats of interventions delivered in each study.

## Abbreviations

EORTC QLQ-C30: European Organisation for Research and Treatment of Cancer Quality of Life Core Questionnaire
FACT-G: Functional Assessment of Cancer Therapy-General
ICT: information and communication technology
PRISMA: Preferred Reporting Items for Systematic Reviews and Meta-Analyses
RCT: randomized controlled trial
