# Supplementary material for: Telemedicine Use Among Caregivers of Cancer Patients: Systematic Review
Source: J Med Internet Res. 2018 Jun 18;20(6):e223. doi: 10.2196/jmir.9812 (PMC6028768; doi:10.2196/jmir.9812)
Supplement: Multimedia Appendix 1 [file jmir_v20i6e223_app1.pdf]

Fig. 1

SEARCH STRATEGY FOR SCIENTIFIC LITERATURE

Search Engine:	Search String:	Hits	Relevant[a]	Included[b]
Pubmed	(((cancer*[Title/Abstract] OR oncolog*[Title/Abstract] OR neoplas*[Title/Abstract] OR tumour*[Title/Abstract] OR tumor*[Title/Abstract] OR carcinoma*[Title/Abstract])) AND (telemedicine[Title/Abstract] OR ehealth[Title/Abstract] OR "e-health"[Title/Abstract] OR mhealth[Title/Abstract] OR "m-health"[Title/Abstract] OR telehealth[Title/Abstract] OR "web based intervention"[Title/Abstract] OR "web based interventions"[Title/Abstract])) AND (caregiver*[Title/Abstract] OR family[Title/Abstract] OR families[Title/Abstract] OR "family member"[Title/Abstract] OR "family members"[Title/Abstract] OR carer*[Title/Abstract])	97	28	8
Google Scholar	(TITLE-ABS-KEY (caregiver OR family OR family AND member OR carers)) AND (TITLE-ABS-KEY (cancer OR neoplas OR tumour OR carcinoma OR oncology)) AND (TITLE-ABS-KEY (ehealth OR mhealth OR telemedicine OR web AND based AND intervention OR telehealth))	227	103	25
Scopus	(TITLE-ABS-KEY (caregiver* OR family OR families OR family AND member* OR carer*)) AND (TITLE-ABS-KEY (cancer* OR neoplas* OR tumour* OR tumor* OR carcinoma* OR oncolog*)) AND (TITLE-ABS-KEY (ehealth OR mhealth OR telemedicine OR web AND based AN D intervention* OR telehealth))	29	12	12
Web of Science	(TOPIC: (caregiver*) OR TOPIC: (family) OR TOPIC: ("family member") OR TOPIC: (carer*)) AND (TOPIC: (telemedicine) OR TOPIC: (ehealth) OR TOPIC: (mhealth) OR TOPIC: (telehealth) OR TOPIC: ("web based intervention")) AND (TOPIC: (cancer) OR TOPIC: (neoplasm) OR TOPIC: (carcinoma) OR TOPIC: (tumour) OR TOPIC: (oncology))	94	13	7
Cochane Library	(("caregiver":ti,ab,kw or "family member":ti,ab,kw or "family":ti,ab,kw or "carer":ti,ab,kw) AND ("telemedicine":ti,ab,kw or "ehealth":ti,ab,kw or "mhealth":ti,ab,kw or "telehealth":ti,ab,kw or "web based intervention":ti,ab,kw) AND ("cancer":ti,ab,kw or "oncology":ti,ab,kw or "carcinoma":ti,ab,kw or "tumour":ti,ab,kw or "neoplasm":ti,ab,kw))	60	20	6

CINAHL	((AB telemedicine OR AB ehealth OR AB mhealth OR AB telehealth OR AB "web based intervention") AND (AB caregiver OR AB "family member" OR AB family OR AB carer) AND (AB cancer OR AB oncology OR AB neoplasm OR AB carcinoma OR AB tumour))	46	15	5
Embase	(('caregiver':ti,ab,kw OR 'family member':ti,ab,kw OR 'family':ti,ab,kw OR 'carer':ti,ab,kw) AND ('telemedicine':ti,ab,kw OR 'ehealth':ti,ab,kw OR 'mhealth':ti,ab,kw OR 'telehealth':ti,ab,kw OR 'web based intervention':ti,ab,kw) AND ('cancer':ti,ab,kw OR 'oncology':ti,ab,kw OR 'carcinoma':ti,ab,kw OR 'neoplasm':ti,ab,kw OR 'tumour':ti,ab,kw))	59	35	3
Ovid PsycINFO	((telemedicine or ehealth or mhealth or telehealth or "web based intervention") and (caregiver* or family or family member or carer*) and (cancer or oncology or neoplasm or tumour or carcinoma)).ab.	43	16	6
**Subtotal**		655	242	72
**Duplicates**				48
**Total**		655	242	24

[a] Relevant: number of relevant articles based on title, abstract, and keywords

[b] Included: number of included articles based on full article
