# Supplementary material for: Telemedicine Use Among Caregivers of Cancer Patients: Systematic Review
Source: J Med Internet Res. 2018 Jun 18;20(6):e223. doi: 10.2196/jmir.9812 (PMC6028768; doi:10.2196/jmir.9812)
Supplement: Multimedia Appendix 2 [file jmir_v20i6e223_app2.pdf]

Caregiver sample, study design and measured outcomes

First author, date, reference	Study country	One site/ multisite	Sample	Study design	Outcome measure	Questionnaires
Shaw, 2016 [62]	Australia	Multicenter	N=128<br>gastrointestinal	RCT<br>Two arms:<br>1) Family Connect telephone intervention (4 calls in 10 weeks after patient hospital discharge)<br>2) usual care<br><br>ASSESSMENT:<br>3 and 6 months	- Quality of Life (QOL)<br>- Caregiver Burden<br>- Unmet supportive care needs<br>- Distress	- SF12<br>- Partner and Caregiver Supportive Care Needs Survey<br>- Caregiver Reaction Assessment (disrupted schedule, financial problems, lack of family support, health impact and impact on self-esteem scales)<br>- Distress Thermometer
Dionne-Odom, 2016 [57]	USA	Multicenter	N=122<br>lung, gastrointestinal, genitourinary, hematologic	RCT<br>Two arms:<br>1) Early group: ENABLE III intervention within 60 days of diagnosis<br>2) Delayed group: 12 weeks later<br><br>ASSESSMENT: By telephone At baseline, 6, 12, 18, and 24 weeks.<br>After Week 24, data collection every 12 weeks until patient's death, or study completion	- Depression<br>- Grief	- CES-D<br>- PG13
Mosher, 2016 [69]	USA	Multicenter	N=106<br>lung	RCT<br>Two arms:<br>1) TSM group<br>2) Education/support group<br><br>ASSESSMENT:<br>4 weekly 45-minute telephone sessions<br>2 and 6 weeks post-intervention	- Anxiety<br>- Confidence in their ability to help the patient manage symptoms<br>- Caregivers' self-efficacy for managing their own emotions<br>- Constraints on cancer-related disclosure from the other dyad member<br>- Caregiver burden	- Generalized Anxiety Disorder seven-item scale (GAD-7)<br>- 16-item standard self-efficacy scale<br>- 8-item self-efficacy scale<br>- five-item social constraints scale<br>- Caregiver Reaction Assessment (impact on schedule, caregiver's esteem, lack of family support, impact on health, and impact on finances)

Dionne-Odom, 2015 [56]	USA	Multicenter	N=122<br>lung, gastrointestinal, breast, hematologic, genitourinary	RCT<br>Two arms:<br>1) Early (fast track)<br>2) Delayed (3 months after diagnosis)<br><br>ASSESSMENT: by telephone once every 6 weeks until week 24 and then every 3 months until patient's death or study completion	- Quality of Life<br>- Depression<br>- CG burden	- CG QOL Scale–Cancer (CQOL-C)<br>- Center for Epidemiologic Study–Depression (CESD) Scale<br>- 14-item Montgomery–Borgatta CG Burden (MBCB) Scale
Chambers, 2014 [71]	Australia	Multicenter	N=336<br>lung, gastrointestinal, breast, hematologic, genitourinary	RCT<br>Two arms:<br>1) Single-session nurse-led self-management intervention<br>2) 5-session psychologist cognitive behavioural intervention<br><br>ASSESSMENT:<br>Baseline (pre-intervention), 3, 6, and 12 months after recruitment	- Distress<br>- Post-traumatic growth	- Brief Symptom Inventory-18 (BSI-18)<br>- Impact of Events Scale (IES)<br>- Posttraumatic Growth Inventory (PTGI)
DuBenske, 2014 [51]	USA	Multicenter	N=246<br>lung	RCT<br>Two arms:<br>1) CHESS + Clinician Report group<br>2) Internet group<br><br>ASSESSMENT:<br>- Intervention time: up to 2 years, or 13 months after patient death<br>- Post-test surveys mailed to caregivers: every 2 months during the period of intervention	- Disruptiveness<br>- Caregiver Burden<br>- Negative mood<br>- Caregiver perception of patient's symptom distress	- Caregiver Quality of Life – Cancer Scale (CQOLC) Disruptiveness Subscale<br>- Caregiver Quality of Life – Cancer Scale (CQOLC) Burden Subscale<br>- Short Version Profile of Mood States (SV-POMS)<br>- Edmonton Symptom Assessment Scale (ESAS)
Kinney, 2014 [49]	USA	Multicenter	N=481<br>colorectal	RCT<br>Two arms:<br>1) TeleCARE group<br>2) Control group<br><br>ASSESSMENT: by telephone Baseline, 1 and 9 months after intervention	- colonoscopy use within 9 months after the intervention	- Risk Behaviour Diagnosis (RBD) Scale

Northouse, 2014 [68]	USA	Multicenter	N=38<br>lung,<br>breast,<br>colorectal,<br>prostate	Feasibility study<br>All participants received the intervention<br><br>ASSESSMENT:<br>Web-based program: 3 sessions over a 6 weeks period, each session every 2 weeks to enable patient-caregiver dyads to learn information in a sequential manner<br><br>T1 (baseline): web-based questionnaires. Dyads logged on to the program and completed sessions together.<br>T2: Two weeks after finishing all three sessions, post-intervention questionnaires	Primary outcomes:<br>- Emotional distress<br>- Quality of life<br><br>Secondary outcomes:<br>- Appraisal<br>- Coping resources<br>- Social support<br>- Self-efficacy	- Profile of Mood States (POMS) (short form)<br>- Functional Assessment of Cancer Therapy (FACT-G version 4)<br>- Benefits of Illness Scale<br>- Communication:Lewis Mutuality and Interpersonal Sensitivity Scale<br>- Brief version of the Social Support Scale<br>- Lewis Cancer Self-efficacy Scale
Badger , 2013 [70]	USA	One center	N=70<br>breast	RCT<br>Two arms:<br>1) Telephone interpersonal counseling (TIP-C)<br>2) Telephone health education (THE)<br><br>ASSESSMENT:<br>8-week intervention<br>At baseline, immediately after the intervention completion (8-week) and 8-week after completion	- Depression<br>- Negative Affect<br>- Stress<br>- Anxiety<br>- Fatigue<br>- Psychological distress<br>- Social well-being<br>- Spiritual well-being<br>- Cancer Knowledge<br>- Social Support	- 20-item Center for Epidemiological Studies-Depression Scale (CES-D)<br>- 10-item negative affect subscale of the Positive and Negative Affect Schedule<br>- Perceived Stress Scale<br>- STAI<br>- Multidimensional Fatigue Inventory<br>- General Symptom Distress Scale<br>- 8-item social well-being subscale of the Quality of Life Breast Cancer<br>- 8-item spiritual well-being subscale of the Quality of Life Breast Cancer instrument<br>- 20-item cancer knowledge measure developed for this study<br>- Perceived Social Support—Family scale

Badger , 2013 [60]	USA	One center	N=52<br>breast	RCT<br>Three arms:<br>1) Telephone interpersonal counseling (TIP-C)<br>2) Telephone health education (THE)<br>3) Videophone interpersonal counselling (VC)<br><br>ASSESSMENT: By telephone<br>- T1: Baseline<br>- T2: intervention completion (after 8-week)<br>- T3: after 8-week from T2<br><br>Intervention: 4 sessions in total, every 2 weeks	- Depression<br>- Symptom distress<br>- Social well-being<br>- Spiritual well-being<br>- User satisfaction	- Center for Epidemiological Studies-Depression Scale (CES-D)<br>- General Symptom Distress Scale (GSDS)<br>- 9-item social well-being scale<br>- 7-item spiritual well-being scale
Clark, 2013 [67]	USA	One center	N=131<br>gastrointestinal,<br>brain,<br>head/neck,<br>lung	RCT<br>Two arms:<br>1) structured multidisciplinary intervention arm<br>2) standard medical care (appointments, referrals to specialists)<br><br>ASSESSMENT:<br>T1: baseline<br>T2; T1 + 4 weeks<br>T3: T1 + 27 weeks	- Quality of Life	- Caregiver Quality of Life Index-Cancer Scale
Chih, 2013 [55]	USA	Multicenter	N=235<br>lung,<br>breast,<br>prostate	RCT<br>Two arms:<br>1) CHESS - Only: access to an interactive cancer communication system<br>2) CHESS + Clinician Report: access to an interactive cancer communication system plus an online symptom reporting system (Clinician Report)<br><br>ASSESSMENT:<br>6 and 12 months after intervention	- caregiver negative mood<br>- caregiving burden and preparedness	- Edmonton Symptom Assessment System (ESAS)<br>- Eastern Cooperative Oncology Group Performance Status<br>- 4-item caregiver Preparedness scale, a subscale of Family Care Inventory<br>- 4-item caregiver Physical Burden scale, a subscale of the Caregiver Burden Inventory<br>- subset of negative mood items from the Shortened Version Profile of Mood States (SV-POMS)

Northouse, 2013 [59]	USA	Multicenter	N=302<br>lung,<br>colorectal,<br>breast,<br>prostate	RCT<br>Three arms:<br>1) Brief (3-session)<br>2) Extensive (6-session) intervention<br>3) Control group (usual care)<br><br>ASSESSMENT:<br>Baseline: prior to intervention<br>Post-intervention: 3 and 6 months from baseline	Primary outcome:<br>- Quality of Life<br><br>Intermediary outcomes:<br>- Appraisals of illness/caregiving<br>- Uncertainty<br>- Hopelessness<br>- Coping<br>- Healthy Behaviour<br>- Interpersonal relationships<br>- Self-efficacy<br>- Communication<br>- Dyadic support<br>- Risk for distress	- Functional Assessment of Cancer Treatment (FACT-G)<br>- Appraisal of Illness or Appraisal of Caregiving Scales<br>- Mishel Uncertainty in Illness Scale<br>- Beck Hopelessness Scale<br>- Brief Coping Orientations to Problems Experienced scale<br>- Scale to assess activities (e.g., exercise, nutrition, adequate sleep)<br>- Lewis Cancer Self-efficacy Scale<br>- Lewis Mutuality and Interpersonal Sensitivity Scale<br>- Social Support Questionnaire<br>- 77-item RFD
Hogberg, 2013 [63]	Sweden	One center	N=6<br>haematological	Feasibility study<br><br>ASSESSMENT:<br>Individual interviews were conducted 4 months after recruitment	- experiences of having access to the web-based communication function<br>- Question of what prevented or allowed the use of web-based communication for support	The interviews were analysed by a content analysis
Scott, 2013 [50]	Australia	One center	N=13<br>early stage cancer	Feasibility study<br><br>ASSESSMENT: by semi-structured interviews.<br>6 modules (released sequentially each week)<br>Post-treatment:1 week<br>Follow-up: 3 months after programme completion	- Negative affect<br>- Distress<br>- Quality of Life<br>- Programme engagement<br>- Qualitative feedback	- 21-item Depression Anxiety Stress Scale<br>- 17-item Posttraumatic Stress Disorder Symptom Scale-Self Report<br>- EORTC QLQ-C30: Functional subscales of Physical, Emotional, Social, Role and the Global QOL scale
Stern, 2012 [48]	Canada	One center	N=12<br>breast,<br>lung,<br>bowel,<br>prostate,<br>kidney,<br>brain	Feasibility study<br><br>ASSESSMENT:<br>Length of exposure to home telehealth: 3.5 months (range: 2 weeks – 6 months)	A) Enhanced access to care:<br>- Ease of access to a health care professional<br>- Reassurance with visual access to care<br>- Enhanced access to pain and symptom management<br><br>B) Usability of the home telehealth system:<br>- Lack of integration of nursing services<br>- Inappropriate timing of the	Qualitative analysis conducted on<br>- data from interviews (caregivers, patients and tele-nurses)<br>- direct observations (families engaged in video-based communication with tele-nurses)<br>- computerized nursing documentation.<br><br>All interviews were audio recorded and transcribed verbatim

					intervention<br>- Lack of portability of the equipment<br>- Technical challenges<br>- Ease of use of the equipment	
Namkoong, 2012 [58]	USA	Multicenter	N=285<br>lung	RCT<br>Two arms:<br>1) CHESS group: access to the CHESS website as well as a laptop computer and Internet access.<br>2) Control group: usual care, a laptop computer with Internet access if needed, and a list of high-quality patient-directed lung cancer and palliative care websites (e.g., cancer.gov and alcase.org) based on clinician recommendations.<br><br>ASSESSMENT:<br>Pre-test surveys prior to randomization<br>Post-test surveys: every 2 months after receipt of the intervention for two years	- Caregiver comfort using the Internet<br>- Caregiver perception of patient's symptom distress<br>- Bonding:universality, group cohesiveness, informational and emotional support<br>- Appraisal and Problem-focused Coping Strategies<br>- Active behavioural coping<br>- Seeking instrumental support	- 5-item bonding scale<br>- Brief Cope questionnaire
Schover, 2012 [53]	USA	One center	N=186 prostate	RCT<br>Three arms:<br>1) [3-month waitlist (WL)]<br>2) 3-session face-to-face format (FF)<br>3) an internet-based format with email contact with the therapist (WEB1)<br>4) second internet-based group (WEB2) was added to further examine the relationship between web site usage and outcomes<br><br>ASSESSMENT:<br>At baseline, post-waitlist, post-treatment, and at 3-, 6-, and 12-	- Erectile dysfunction<br>- Female sexual function<br>- Emotional distress<br>- Relationship satisfaction	- International Index of Erectile Function (IIEF)<br>- Female Sexual Function Index (FSFI)<br>- Brief Symptom Inventory (BSI-18)<br>- abbreviated Dyadic Adjustment Scale

				month		
Zulman, 2012 [65]	USA	Multicenter	N=38<br>lung,<br>colorectal,<br>breast,<br>prostate	Feasibility study<br>4 focus group and usability testing.	5 core modules:<br>- Family involvement<br>- Optimistic attitude<br>- Coping effectiveness<br>- Uncertainty reduction<br>- Symptom management	Transcripts of the recorded groups were analysed by two members of the content team to identify themes and representative quotes.
Badger, 2011 [64]	USA	Multicenter	N=70<br>prostate	RCT<br>Two arms:<br>1) Telephone interpersonal counselling (TIP-C)<br>2) Health education attention condition (HEAC)<br><br>ASSESSMENT: By telephone<br>T1: Baseline<br>T2: T1 + 8 weeks<br>T3: T2 + 8 weeks<br><br>Intervention:<br>4 calls in total, every 2 weeks	- Depression<br>- Positive and Negative Affect<br>- Symptom distress<br>- Fatigue<br>- Social well-being<br>- Spiritual well-being<br>- Social Support	- 20-item Center for Epidemiological Studies-Depression Scale (CES-D)<br>- 20-item Positive and Negative Affect Schedule<br>- Perceived Stress Scale (PSS)<br>- Multidimensional Fatigue Inventory<br>- 8-item social well-being modified subscale of the Quality of Life Breast Cancer<br>- 8-item spiritual well-being subscale of the modified Quality of Life Breast Cancer instrument<br>- Perceived Social Support—Family scale (PSS-FA)
Porter, 2011 [61]	USA	Multicenter	N=233<br>lung	RCT<br>Two arms:<br>1) Caregiver-assisted Coping skills training group (CST)<br>2) Education/support group<br><br>ASSESSMENT: By telephone<br>T1: Baseline<br>T2: post-treatment immediately after intervention's completion<br>T3: T2 + 4-month<br><br>Intervention:<br>14 telephone-based sessions	- psychological distress<br>- caregiver strain<br>- self-efficacy for helping the patient manage symptoms	- Profile of Mood States-B (POMS-B)<br>- Caregiver Strain Index (CSI)<br>- Chronic Pain Self-Efficacy scale (modified version)

Northouse, 2007 [52]	USA	Multicenter	N=235 prostate	RCT<br>Two arms:<br>1) FOCUS program group<br>2) Usual care<br><br>Assessment:<br>Baseline, and 4, 8, 12 months after intervention's completion	- Quality of life<br>- Appraisals of illness/caregiving<br>- Uncertainty<br>- Hopelessness<br>- Coping strategies<br>- Self-efficacy<br>- Communication<br>- General symptom distress<br>- Extent to which husbands' prostate-specific symptoms created problems for spouses<br>- Risk for distress	- Medical Outcomes Study 12-item short form (MOS SF-12)<br>- Functional Assessment of Cancer Treatment (FACT-G)<br>- Appraisal of Illness or Appraisal of Caregiving Scales<br>- Mishel Uncertainty in Illness Scale<br>- Beck Hopelessness Scale<br>- Brief Coping Orientations to Problems Experienced scale<br>- Lewis Cancer Self-Efficacy Scale<br>- Lewis Mutuality and Interpersonal Sensitivity Scale<br>- Omega Screening Questionnaire (OSQ)<br>- 4-item spousal version of the EPIC<br>- 77-item RFD
Northouse, 2005 [54]	USA	Multicenter	N=134 breast	RCT<br>Two arms:<br>1) FOCUS program group<br>2) Usual care<br><br>ASSESSMENT:<br>Baseline, 3-months after initial phase completion, and 6-months after booster phase completion of the program	- Appraisal of illness<br>- Appraisal of caregiving<br>- Uncertainty<br>- Hopelessness<br>- Coping<br>- Quality of life	- Appraisal of Illness Scale<br>- Appraisal of Caregiving Scale<br>- Uncertainty in Illness Scale<br>- Beck Hopelessness Scale<br>- Brief COPE<br>- Functional Assessment of Cancer Treatment (FACT-G)<br>- SF-36
Farnham, 2002 [66]	USA	One center	N=49<br>bone marrow transplant	RCT<br>Two arms:<br>1) HutchWorld group: computers with access to the Internet and HutchWorld<br>2) Control group: no access to the Internet in their apartments<br><br>ASSESSMENT:<br>Baseline, monthly and weekly questionnaires for up to 3 months + debriefing session at the end of the study (feedback on HutchWorld and the study	- Quality of Life<br>- Social support<br>- Internet and web-based program usage patterns	Not specified

				itself)<br>Participants submitted their questionnaires using a self-addressed envelope		

Abbrevations

BSI-18: Brief Symptom Inventory-18

CESD: Center for Epidemiological Studies- Depression scale

CHESS: Comprehensive Health Enhancement Support System

CQOL-C: Caregiver Quality of Life Index-Cancer scale

CSI: Caregiver Strain Index

CST: Caregiver-assisted Coping skills training group

ENABLE: Educate Nurture Advise Before Life Ends

EORTC QLQ-C30: European Organisation for Research and Treatment of Cancer Quality of Life Core Questionnaire

ESAS: Edmonton Symptom Assessment Scale

FSFI: Female Sexual Function Index

HEAC: Health education attention condition

GAD-7: Generalized Anxiety Disorder seven-item scale

GSDS: General Symptom Distress Scale

IES: Impact of Events Scale

IIEF: International Index of Erectile Function

MBCB: Montgomery–Borgatta CG Burden scale

MOS SF-12: Medical Outcomes Study 12-item short form

OSQ: Omega Screening Questionnaire

PG13: Prolonged Grief scale

PSS: Perceived Stress Scale

SV-POMS: Shortened Version Profile of Mood States

PTGI: Posttraumatic Growth Inventory

RBD: Risk Behaviour Diagnosis Scale

RCT: randomized controlled trial

RFD: Risk for Distress scale

SF 12: 12-Item Short Form Survey

STAI: State-Trait Anxiety Inventory

SV-POMS: Short Version Profile of Mood States

TIP-C: Telephone interpersonal counseling

THE: Telephone health education

TSM: Telephone-based Symptom Management
