# Supplementary material for: Telemedicine Use Among Caregivers of Cancer Patients: Systematic Review
Source: J Med Internet Res. 2018 Jun 18;20(6):e223. doi: 10.2196/jmir.9812 (PMC6028768; doi:10.2196/jmir.9812)
Supplement: Multimedia Appendix 3 [file jmir_v20i6e223_app3.pdf]

YEAR	AUTHORS	PROJECT	INTERVENTION TYPE AND CHARACTERISTICS
2016	Shaw et al.	FAMILY CONNECT	TELEPHONE SESSIONS + WRITTEN MATERIALS<br><br>- Manualized and standardized assessment of caregiver need across the domains of patient care (emotional, physical self-care, information).<br>- List of resources and strategies<br>- Individualized information related to local patient and/or caregiver support services and practical and financial resources available for caregivers
2016	Dionne-Odom et al.	ENABLE III	TELEPHONE SESSIONS + WRITTEN MATERIAL<br><br>- Manualized intervention by nurse coaches over the phone.<br>- Charting Your Course–Caregiver (CYC-CG) by email: guidebook of resource material supporting the nurse coaching sessions.<br>- 3-weekly telephone coaching sessions using CYC-CG:<br>o Session 1: “Being a caregiver”: goals of palliative care, coping and problem-solving attitude<br>o Session 2: “Caregiver self-care”: healthy eating, exercise, meditation, spirituality, and patient's symptom management<br>o Session 3: communication, decision making, advance care planning.<br>- monthly calls to reinforce and assist new issues
2016	Moscher et al.	Telephone Symptom Management Intervention	TELEPHONE SESSIONS + WRITTEN MATERIAL<br>Patients and their caregivers participated simultaneously through speakerphone<br><br>TMS group:<br>- symptom management strategies by evidence-based cognitive-behavioral and emotion-focused strategies for managing anxiety and depressive symptoms, pain, fatigue, and breathlessness<br>- written material on discussed major points and home practice assignments<br>- CD with instructions for relaxation exercises.<br><br>Education/Support group:<br>- orientation to the medical center and treatment team<br>- psychoeducation<br>- resources for health information, psychosocial support, and financial concerns<br>- Evaluating health information on the Internet
2015	Dionne-Odom et al.	ENABLE III	TELEPHONE SESSIONS + WRITTEN MATERIAL<br><br>- Manualized intervention by nurse coaches over the phone.<br>- Charting Your Course–Caregiver (CYC-CG) by email: guidebook of resource material supporting the nurse coaching sessions.<br>- 3-weekly telephone coaching sessions using CYC-CG:<br>o Session 1: “Being a caregiver”: goals of palliative care, coping and problem-solving attitude<br>o Session 2: “Caregiver self-care”: healthy eating, exercise, meditation, spirituality, and patient's symptom management<br>o Session 3: communication, decision making, advance care planning.<br>- monthly calls to reinforce and assist new issues
2014	Chambers et al.	Low-intensity Psychological Interventions	TELEPHONE SESSIONS + WRITTEN MATERIAL+AUDIO CD<br><br>NURSE SELF MANAGEMENT<br>- Single-session of psychoeducation<br>- Audio CD with relaxation exercises<br><br>PSYCHOLOGIST-DELIVERED COGNITIVE BEHAVIORAL<br>- Psychoeducation

			- Problem-solving<br>- Cognitive therapy<br>- Enhancement of support network<br>- Behavioural homework
2014	DuBenske et al.	CHESS	EHEALTH SYSTEM: “Coping with Lung Cancer: A Network of Support”<br><br>CHESS Group:<br>- access to ready and organized access to information<br>- channel for communication and support with peers, experts, and users’ social networks<br>- coaching services<br><br>Internet Group:<br>- Standard care<br>- Computer with internet access (if needed)<br>- List of lung cancer and palliative care websites based
2014	Kinney et al.	FAMILY Care	TELEPHONE SESSIONS + WRITTEN MATERIAL<br><br>TeleCARE group:<br>- educational brochure and tailored visual aids<br>- sessions tailored based on the participant's responses to key items assessed at baseline<br>- telephone session on risk communication and behavior change approaches<br>- client style of motivational interviewing<br>- all sessions audio-taped<br>- final summarizing tailored letter immediately after the telephone session<br><br>Control Group:<br>- educational brochure
2014	Northouse et al.	FOCUS Program	EHEALTH SYSTEM<br>Web-based format of “Family Involvement Module”:<br><br>- tailored messages based on information provided at baseline and during the web-based sessions<br>- tailored variables<br>- tailored strategies: personalization, tailored feedback, content matching<br>- tailored links to other websites<br>- tailored activities to promote dyadic interactions<br><br>Dyads completed sessions together, sitting side-by-side at the computer.
2013	Badger et al.	Telephone-delivered health education and interpersonal counselling	TELEPHONE SESSIONS<br><br>TIP-C intervention: counselling on<br>- mood and affect management<br>- emotional expression<br>- interpersonal communication and relationships<br>- social support<br>- cancer information<br><br>THE intervention: education on<br>- normal vs disease status, routine tests, prevention and associated terminology<br>- treatment, side effects and strategies to combat<br>- lifestyle interventions (nutrition, physical activity)<br>- referrals and resources

2013	Badger et al.	Telephone-delivered health education and interpersonal counselling	TELEPHONE SESSIONS<br><br>TIP-C intervention and VC: counselling on<br>- mood and affect management<br>- emotional expression<br>- interpersonal communication and relationships<br>- social support<br>- cancer information<br>THE intervention: education on<br>- normal vs disease status, routine tests, prevention and associated terminology<br>- treatment, side effects and strategies to combat<br>- lifestyle interventions (nutrition, physical activity)<br>- referrals and resources
2013	Clark et al.	Structured Multidisciplinary Intervention on maintaining Quality of Life	IN-PERSON INTERVENTION + TELEPHONE SESSIONS<br><br>6 Structured multidisciplinary sessions, caregivers attended sessions 1 (Health behavior changes), 3 (Coping with cancer), 4 (Social needs), and 6 (Communication strategies)<br><br>10 brief telephone session on:<br>- Review of self-care<br>- Mood management skills<br>- Spiritual needs<br>- Physical therapy<br>- Health behavior changes<br>- Communication and social support<br>- Social needs<br>- Mood assessment<br>- Physical therapy
2013	Chih et al.	CHESS	EHEALTH SYSTEM<br><br>CHESS System:<br>- access to ready and organized access to information<br>- channel for communication and support with peers, experts, and users' social networks<br>- coaching services<br><br>Both groups received CHESS access, but clinicians of CHESS+CR group received reports before clinic visit
2013	Northouse et al.	FOCUS Program	HOME VISITS INTERVENTION + TELEPHONE SESSIONS<br><br>Content of both programs was the same:<br>- family involvement<br>- optimistic attitude<br>- coping effectiveness<br>- uncertainty reduction<br>- symptom management<br><br>Brief PROGRAM: 2 home visits and 1 phone call<br>Extensive PROGRAM: 4 home visits and 2 phone sessions
2013	Hogberg et al.	web-based communication for psychosocial support	EHEALTH SYSTEM<br><br>Web based format with 2 sections:<br>1) "My care contacts": to change bookings and renew prescriptions<br>2) "Psychosocial support": information, advice and guidance for need conditions
2013	Scott & Beatty	Self-guided cognitive behaviour	EHEALTH SYSTEM<br><br>6-week internet cognitive behaviour therapy (CBT) programme:<br>- starting treatment

		therapy Internet intervention	- coping with physical symptoms and side effects<br>- coping with emotional distress<br>- body image, identity and sexuality<br>- your family and friends<br>- completing treatment<br><br>Each module provides psycho-education, cognitive-behavioural worksheets / strategies, and survivor stories and quotes + personal blog for online-journal writing, and a resources section.
2012	Stern et al.	Home Telehealth	TELEPHONE SESSIONS<br><br>Nurses with expertise in palliative care and available 24 hours per day communicated with patients and family caregivers via telephone or videophone
2012	Namkoong et al.	CHESS	EHEALTH SYSTEM<br><br>Computer-mediated social support group offering text-based, asynchronous bulletin boards on information, communication, and coaching resources<br><br>CHESS modules:<br>- Information<br>- Support<br>- Tools
2012	Schover et al.	CAREss	EHEALTH SYSTEM + TELEPHONE INTERVENTIONS<br><br>same content and cognitive-behavioral homework:<br>- standardized homework exercises completed on paper<br>- therapists discussed homework (FF) in session or emailed feedback to the couple and can be contacted at any time (WEB)<br>- therapist's exercises to increase expression of affection, improve sexual communication, increase comfort in initiating sexual activity, and facilitate resuming sex without performance anxiety (using a sensate focus framework)<br>Booster phone calls were made at 1 and 3 months to discuss progress and ways to overcome remaining barriers
2012	Zulman et al.	FOCUS Program	EHEALTH SYSTEM<br><br>Contents:<br>- family involvement<br>- optimistic attitude<br>- coping effectiveness<br>- uncertainty reduction<br>- symptom management
2011	Badger et al.	Telephone-delivered health education and interpersonal counselling	TELEPHONE SESSIONS<br><br>TIP-C intervention: counselling on<br>- mood and affect management<br>- emotional expression<br>- interpersonal communication and relationships<br>- social support<br>- cancer information<br><br>HEAC group: written materials about<br>- prostate cancer diagnosis and treatments<br>- health-related topics (eg. nutrition, exercise to decrease fatigue, resources for cancer survivors, and quitting smoking)
2011	Porter et al.	Coping Skills Training	TELEPHONE SESSIONS<br><br>Sessions 1–7. Training in specific coping skills:

			- progressive muscle relaxation<br>- pleasant imagery relaxation<br>- activity pacing method<br>- cognitive restructuring<br>- problem solving strategies<br>- goal setting and pleasant activity scheduling<br>- effective communication of thoughts and feelings<br><br>Sessions 8–12. Training in alternative relaxation, imagery exercises, and application of the coping<br><br>Sessions 13-14. Maintenance strategies<br><br>The nurse used a 3-step behavioral rehearsal procedure: the dyad practiced the skill together, and the nurse provided feedback to the dyad on their practice.
2007	Northouse et al.	FOCUS Program	HOME VISITS + TELEPHONE INTERVENTIONS<br><br>Contents:<br>- family involvement<br>- optimistic attitude<br>- coping effectiveness<br>- uncertainty reduction<br>- symptom management<br><br>FOCUS Program: 3 home visits and 2 phone sessions spaced 2 weeks
2005	Northouse et al.	FOCUS Program	HOME VISITS + TELEPHONE INTERVENTIONS<br><br>Contents:<br>- family involvement<br>- optimistic attitude<br>- coping effectiveness<br>- uncertainty reduction<br>- symptom management<br><br>FOCUS Program: 3 home visits and 2 phone sessions spaced 2 weeks
2002	Farnham et al.		EHEALTH SYSTEM<br><br>HutchWorld: web portal with<br>- tools for social interactions, access to information, and diversionary activities<br>- variety of solo or group activities<br>- customization of user’s graphical representation<br>- forum where questions could be posted and answered<br>- chat and gesture to one another, exchange gifts, play games, or explore to environment<br>- integrating email, relevant web sites, journaling and other activities increased the ease of accessing key information
